# A socio-ecological exploration to identify factors influencing the COVID-19 vaccine decision-making process among pregnant and lactating women: Findings from Kenya

**DOI:** 10.1016/j.vaccine.2022.10.068

**Published:** 2022-11-28

**Authors:** Rupali J. Limaye, Alicia Paul, Rachel Gur-Arie, Eleonor Zavala, Clarice Lee, Berhaun Fesshaye, Prachi Singh, Wincate Njagi, Paul Odila, Paul Munyao, Rosemary Njogu, Stephen Mutwiwa, Lisa Noguchi, Christopher Morgan, Ruth Karron

**Affiliations:** a Department of International Health, Johns Hopkins University, Bloomberg School of Public Health, Baltimore, MD, USA; b International Vaccine Access Center, Johns Hopkins University, Bloomberg School of Public Health, Baltimore, MD, USA; c Department of Health, Behavior & Society, Johns Hopkins University, Bloomberg School of Public Health, Baltimore, MD, USA; d Department of Epidemiology, Johns Hopkins University Bloomberg School of Public Health, Baltimore, MD, USA; e Berman Institute of Bioethics, Johns Hopkins University, Bloomberg School of Public Health, Baltimore, MD, USA; f Jhpiego, Kenya; g Jhpiego, Baltimore, MD, USA

**Keywords:** Maternal immunization, COVID-19, Pregnant women, Vaccine acceptance, Communication

## Abstract

The vaccine decision-making process of pregnant and lactating women is complex. Regarding COVID-19, pregnant women are at increased risk for severe disease and poor health outcomes. While pregnant and lactating women were excluded from COVID-19 vaccine trials, available evidence suggests that COVID-19 vaccines are safe and protective during pregnancy. In this study, we used a socio-ecological approach to explore factors influencing the decision-making process for COVID-19 vaccines in pregnant and lactating women in Kenya, for the purpose of informing demand generation strategies. As pregnant and lactating women are influenced by many factors, we conducted 84 in-depth interviews with a variety of stakeholders, including 31 pregnant or lactating women, 20 healthcare workers such as nurses, midwives, doctors, and frontline workers, 25 male family members of pregnant or lactating women, and 8 gatekeepers such as community leaders and faith-based leaders. These individuals were recruited from six communities in Kenya: three urban, and three rural. We applied a grounded theory approach to identify emerging themes and organized emerging themes using the SAGE Vaccine Hesitancy model, which includes three categories of determinants of vaccine acceptance, including contextual influences, individual and group influences, and vaccine and vaccination specific issues. Myths, interpersonal norms, and religion emerged as themes related to contextual influences. Safety, risk perception, and the role of the healthcare worker emerged as themes related to individual and group influences. For vaccine and vaccination specific issues, emerging themes included availability, accessibility, and eligibility. While maternal immunization can substantially reduce the effect of infectious diseases in mothers and infants, vaccine acceptance is critical. However, vaccines do not save lives; vaccination does. We hope the results of this study can be used to tailor communication efforts to increase vaccine demand among pregnant and lactating women.

## 1 Introduction

Vaccination is a health decision that impacts the health, well-being, and safety of communities. Vaccine decision-making is complex, and is influenced by a variety of biological, behavioral, clinical, social, environmental, and economic factors [20]. Such factors vary according to the disease targeted by the vaccine, vaccine safety and effectiveness, and the target population [10]. Understanding vaccine decision-making is crucial to promote vaccine uptake and prevent disease outbreaks. Vaccine confidence has been publicly challenged [14], [18]. Investigating and understanding factors that contribute to vaccine confidence and decision-making is crucial to craft ethical and effective vaccine communication and policies that maximize vaccine uptake.

The decision to vaccinate is further complicated during pregnancy. Prior research has established several critical factors which influence pregnant women in the vaccine decision-making process. Thought to be the most influential factor is the recommendation of a health provider [13], [17], [37], given providers’ role as a trusted source [8], [15], [24], [33], [40]. In their systematic review and *meta*-analysis of maternal vaccine decision-making, Kilich et al. [17] found that receiving a provider recommendation increased pregnant women’s odds of vaccine uptake for pertussis or influenza by up to 12 times. Another influential factor in maternal vaccine decision-making is concern surrounding vaccine safety [33], [40]. Pregnant women report fears of vaccine side effects, pain of vaccination, long-term consequences, and harm to the fetus and/or child as common barriers to uptake [22], [23], [26], [33]. Knowledge or perceptions of disease risk as well as vaccine benefits are particularly relevant in maternal vaccine decision-making [19], [37], [40].

Pregnant and lactating women have historically been excluded from most vaccine clinical trials [25]. Most COVID-19 vaccine trials have followed suit, outlining pregnancy and lactation as exclusion criteria [32], despite repeated calls for inclusion of pregnant women in COVID-19 vaccine trials [5]. Reasons for exclusion lie in the safety concerns for both the mother and infant [35].

The combination of post-authorization evidence on the safety of COVID-19 vaccine use in pregnancy and lactation [6], [31], and the epidemiological data demonstrating the increased risks of severe disease and poor birth outcomes due to COVID-19 infection in pregnancy [16], [21], has led global and national public health authorities to recommend the use of COVID-19 vaccines in pregnancy and lactation, and recommendation policies have evolved over the course of the pandemic [39]. Despite these recommendations and the growing body of evidence that the vaccines are safe and protective in pregnancy, pregnant women remain one of the high-risk groups with the lowest uptake of COVID-19 vaccines. In this study, we explored the factors influencing the decision-making process for COVID-19 vaccines in pregnant and lactating women in Kenya to inform future demand generation strategies.

## 2 Methods

This qualitative study conducted in-depth interviews with a diverse set of audiences that may influence the vaccine decision-making process of pregnant or lactating women. Interviews were conducted with pregnant women, lactating women, male family members of pregnant or lactating women, community gatekeepers, and healthcare workers. Participants were recruited from three counties, with two communities in each county: Garissa (rural), Kakamega (rural and urban), and Nairobi (urban).

Data were collected in August-September 2021. Interview instruments were pre-tested with pregnant women living in Kenya. Interview guides included questions related to the COVID-19 vaccine decision-making process for pregnant and lactating women. Data collectors participated in a three-day training exercise, after completing an online human ethics training. Participants were recruited at various health clinics across the six communities. If a participant met the inclusion criteria and agreed to participate, oral consent was obtained. Interviews were conducted in either English, Swahili, or other local languages as necessary in a semi-private setting or via Zoom. All interviews were audio recorded. Members of the study team transcribed and translated the transcripts into English. All data were stored on encrypted servers, and only members of the study team had access to the data. Study activities involving in-person interaction including training and data collection were conducted following COVID-19 safety protocols per the Ministry of Health in Kenya.

A team of seven analyzed the data through a grounded theory approach. The team conducted three rounds of open coding to develop, refine, and finalize a code list. After open coding and agreement of a code list, team members coded 24 transcripts and discussed emerging themes. The team then coded the remaining transcripts. Two members of the team conducted inter-rater reliability with ∼ 10 % of the transcripts that neither of them had coded (10 transcripts). Reliability was > 90 %. The team then identified themes and sub-themes. Data were managed using Atlas.ti. This study received ethical approval from Johns Hopkins Bloomberg School of Public Health and Kenya Medical Research Institute.

## 3 Results

A total of 84 individuals were interviewed, including 31 pregnant or lactating women, 20 healthcare workers, 25 male family members of pregnant or lactating women, and 8 gatekeepers (Table 1).

**Table 1:** Study Participants by Participant Type & Location (n = 84).

	Garissa (rural)	Kakamega (rural and urban)	Nairobi (urban)	**Total**
Pregnant and lactating women	10	10	11	**31**
Healthcare workers (midwives, nurses, doctors, and immunization workers)	6	8	6	**20**
Male family members	6	8	11	**25**
Gatekeepers (faith-based leaders and community leaders)	2	2	4	**8**

**Fig. 1:**
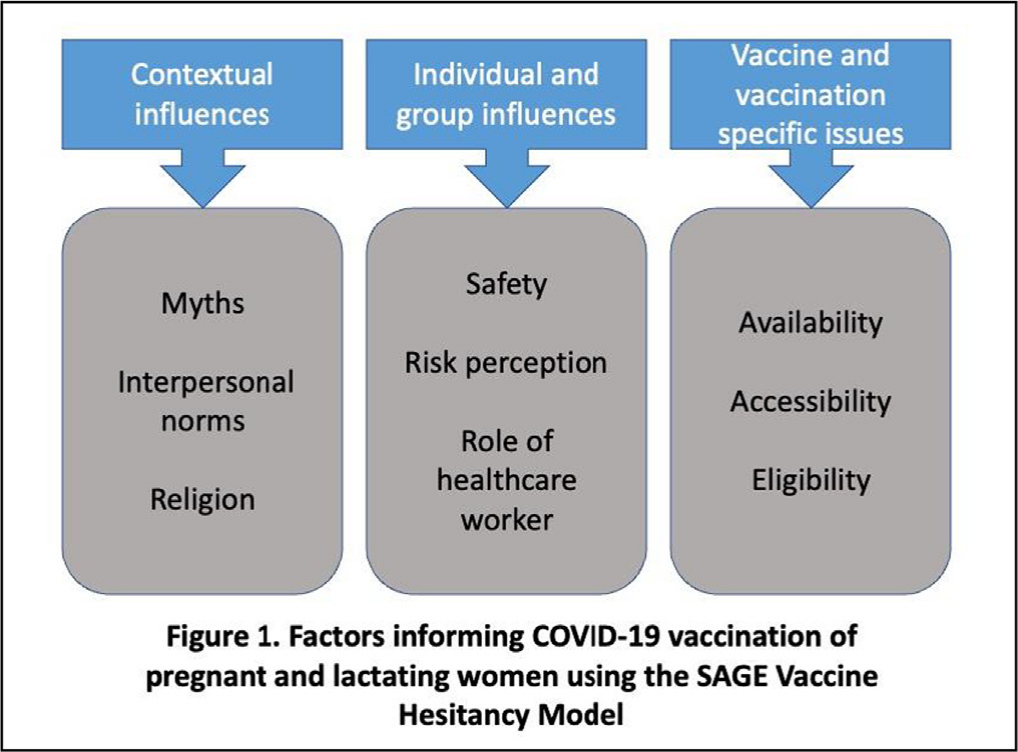
Factors informing COVID-19 vaccination of pregnant and lactating women using the SAGGE vaccine Hesitancy Model.

We organized emerging themes using the SAGE Vaccine Hesitancy model [11]. This model includes three categories of determinants of vaccine acceptance, including contextual influences, individual and group influences, and vaccine and vaccination specific issues. See Fig. 1 for themes classified by this model. Under contextual influences, emerging themes from our study included myths, interpersonal norms, and religion. Related to individual and group influences, safety, risk perception, and the role of the healthcare worker were the themes that emerged. For vaccine and vaccination specific issues, emerging themes included availability, accessibility, and eligibility.

We summarize these emerging themes, comparing and contrasting among the target audiences we interviewed, in the following sections.

### 3.1 Contextual Influences: Myths

Many participants discussed myths and rumors related to COVID-19 vaccines.

Each participant type articulated the various myths they had heard about COVID-19 vaccines related to pregnant and lactating women.

Pregnant women specifically were concerned about what they had heard related to the effects of the vaccine on their pregnancies, such as this pregnant woman from a rural community in Kakamega: “I have heard that if you get the vaccine, you will be numb. You won’t grow fat. You won't have an appetite. You will just be tired, your body won't function.” Several participants indicated that they believed that the vaccine was actually not a vaccine, but something else, including this lactating woman from a rural community in Kakamega: “There are those mothers that say that they can’t go for the vaccine…there are those that are not willing to go for it. They are against it and they feel like it is not the COVID vaccine. They feel that it is something else.”

Healthcare workers spoke about vaccine myths they heard from their pregnant clients, including this healthcare worker from an urban community in Kakamega: “I can say that they have not embraced (the vaccine), they believe that the vaccines have been developed to kill them.” This healthcare worker from a rural community in Kakamega shared a similar sentiment when asked about their pregnant clients: “Some think that it has side effects and others think that it is a vaccine that was brought to kill us. It targets certain groups to kill them, especially the elderly because they think they are a burden and it wants to eradicate them.” Another worker from a rural community in Kakamega raised the issue of the vaccine’s link to infertility and death: “One myth I have heard from clients is that it makes you infertile…there’s also this myth that if given you won't stay long. Given some few years you die.”

Male family members of pregnant and lactating women were most concerned about the link between the vaccine and sexual and reproductive health. This male family member from a rural community in Kakamega illustrated this concern: “You find that people say that it affects libido. If this person is not ready to lose their libido, will you accept such a vaccine? No, you won’t.” This male family member from an urban community in Kakamega discussed why he would not approve of pregnant women getting the vaccine: “Pregnant women, when they are given the vaccine, it can affect them and once they deliver they will never get pregnant again, they will be completely infertile.” Another male family member from an urban community in Kakamega heard that the vaccine was a form of population control: “Some people say it's like the Chinese want to reduce Kenyans.”

Gatekeepers also identified myths related to fertility, as well as other physical ailments. This gatekeeper from a rural community in Garissa said: “Some are saying that the vaccine will stop women from conceiving, a lot of headaches, you will get sick.” This gatekeeper from an urban community in Nairobi mentioned several myths: “Peopl;e say the vaccines are here to kill people and to decimate the population, or will not give birth.”

### 3.2 Contextual Influences: Interpersonal norms

Norms, or what other community members felt about and/or did in relation to the vaccine, were important for pregnant and lactating women. More specifically, male partners were especially influential in informing pregnant and lactating women’s vaccine decision-making process.

Many pregnant and lactating women alluded to the fact that they would want to see what others were doing, or the norms in their community, to help them decide about the vaccine. This pregnant woman from a rural community in Garissa said: “I would ask them (other pregnant women) what they felt after getting the vaccine… did they feel better or did they feel worse because it will determine if I will get the vaccine.” This lactating woman from a rural community in Kakamega said she would consider how others felt to make her decision: “I will consider being educated first and then seeing the number of people that will be vaccinated and if the mass of those are afraid then I will be afraid too.” This lactating woman from a rural community in Kakamega referenced the behaviors of her peers in her community: “They keep on saying that they will go for the vaccine a certain day and when that day comes, they don’t go. If they see anyone having side effects, they will say that they are busy.” This pregnant woman from a rural community in Kakamega referred to the influence of her husband: “My husband has gone for it but he is challenging me that he does not know about expectant mothers.”

Healthcare workers did not indicate that norms were influential in vaccine decision-making among pregnant and lactating women. Male family members asserted their influence on their spouse, as per this male family member from an urban community in Nairobi: “My pregnant wife, she is following what the community is saying but I told her, if the vaccine is being given, then you must receive the vaccination because all public servants are being given.” Gatekeepers similarly identified the influence of the male partner, as illustrated by this gatekeeper from a rural community in Kakamega: “My wife told me, ‘my hubby if you go, we all have to go.’”.

### 3.3 Contextual Influences: Religion

Religion was raised as an important influence of vaccination by healthcare workers and gatekeepers specifically. Both groups alluded to religious teachings as a framework to guide pregnant and lactating women about their decision about the vaccine. Additionally, faith-based gatekeepers articulated their trust in healthcare workers regarding their recommendation.

This healthcare worker from an urban community in Nairobi lamented the challenges of working with churches on vaccinating pregnant and lactating women: “There was a time when the Catholic Church was against vaccines, and it was difficult for us to encourage the Catholic Christians. The vaccination of pregnant mothers… is very important, because it protects the mother and the infant.” This faith-based gatekeeper from a rural community in Garissa asserted that he trusted healthcare workers: “You know I am not a doctor…we follow the doctors’ instructions. Any medication that helps human beings, we, as religious leaders, do not have a problem. The thing that we do not want and cannot accept is vaccination that affects the mother and her child. If that vaccine will affect the mother and the baby in the womb then we religious leaders do not accept that.” This faith-based gatekeeper from an urban community in Nairobi stressed the importance of communication from faith-based leaders: “We need to give accurate information, we need to disseminate. We are trusted… the church is the best agent, because the church is known for training and teaching about the truth.” This faith-based gatekeeper from a rural community in Garissa urged the prioritization of pregnant and lactating women: “I would prioritize breastfeeding mothers and pregnant women. Those two groups are the most vulnerable in society because pregnant women are growing another life inside of them. As for the breastfeeding mothers, according to our religion, they should be treated with kindness.”

### 3.4 Individual & group influences: Safety

Vaccine safety was the most frequently expressed concern related to vaccine decision-making, including perceived safety concerns such as myths. This was found across the target audiences. Safety concerns included immediate effects to the person receiving the vaccine, unknown effects pertaining to the person receiving the vaccine and the unborn child, and long-term effects to the person receiving the vaccine and the unborn child.

Pregnant women had concerns about how the vaccine would affect their health. This pregnant woman from an urban community in Nairobi expressed concern about side effects: “In the community there are people that have been vaccinated, but not pregnant women. There are those that have gone for the vaccines and they have complained of back ache. Some after receiving the vaccine they become sick. I am wondering, ‘if we the pregnant women are to be injected how will it react with us?’” Many of the pregnant women were concerned about the vaccine causing death, such as this pregnant woman from a rural community in Garissa: “There have been many fatalities linked to the vaccine given to people…and serious side effects, so I am not ready to get it.” Lactating women expressed serious side effects as well, such as this lactating woman from an urban community in Nairobi: “Some say they have been vaccinated and they experienced chest pains for two weeks, others have been vaccinated and they can’t function.” Other lactating women were concerned about the unknowns related to the vaccine. This lactating woman from a rural community in Garissa, when asked if she would get the vaccine, said: “We don’t know how the vaccine affects or brings more sickness to the body.” This sentiment - that the side effects of the vaccine were unknown and would inform their decision-making process - was raised by many lactating women, such as this lactating woman from an urban community in Nairobi: “As long it is helping and it does not affect one’s health or the child who is breastfeeding, it is good.”

Healthcare workers expressed several safety concerns they heard from pregnant and lactating women. Several raised blood clots, including this healthcare worker from a rural community in Garissa: “Some people were explaining about the AstraZeneca vaccine causing blood clots. That has also brought a lot of issues to the people who are willing to take the vaccine.” This healthcare worker from an urban community in Nairobi also commented blood clots: “But there is still that fear, there are those people who say that you can be vaccinated and get a blood clot… so that fear, the fact that we know that after vaccination, you can get bleeding disorder, this discourages people.” Healthcare workers also relayed concerns from pregnant women of the negative effects that the vaccine could have on the unborn child. This healthcare worker from a rural community in Kakamega said: “When you give the pregnant women health education, most of them say that the COVID-19 vaccine may cause damage to their unborn children and some fear that they may die because there was rumor that those people who received the vaccine will die in the next two years.”

Male family members of pregnant and lactating women articulated their perceptions of the safety of the vaccine for pregnant women, including this male family member from an urban community in Nairobi: “The pregnant mothers are vulnerable and the vaccine might bring complications to them and I feel it is not right to give them the vaccine.” This male family member from an urban community in Nairobi also spoke about healthcare workers and their concerns about the vaccine: “Most of the healthcare workers are afraid and they say that after someone getting the vaccine there are many side effects.” This male family member from a rural community in Garissa discussed his concern about fertility: “Some say that if you have not given birth chances of not being able to have kids in future is very high.”

Gatekeepers expressed how their fellow community members had concerns about the vaccine. When asked about vaccine acceptance in their community, this gatekeeper from a rural community in Garissa touched upon safety concerns: “It is a very little percentage (that have taken the vaccine) because people fear this vaccine. People are very scared because your blood clots and people have died.” Related to vaccinating pregnant or lactating women, this gatekeeper from an urban community in Nairobi had heard that this group should not be vaccinated due to safety issues: “I heard that the expectant mothers are not to be vaccinated. I generally heard that it’s not safe for them.”

### 3.5 Individual & group influences: Risk perception

When asked about risk perception, each target audience reflected on their own risk perception. Risk perception among pregnant and lactating women was generally high across all counties.

Risk related to the unborn child was raised as a reason why to get the vaccine. As this pregnant woman from a rural community in Kakamega articulated: “Well (the vaccine) is good for you and also benefits the unborn child.” This pregnant woman from a rural community in Garissa mentioned that the vaccine supported full-term birth: “If you get the vaccine, you will carry your baby to full term and you will not be sick.” This lactating woman from an urban community in Nairobi echoed this sentiment about protecting the unborn child: “It is important to get the vaccine because you are avoiding many things from happening to the baby you are carrying.” This pregnant woman from a rural community in Kakamega stressed how the vaccine protects the mother: “For me because I'm a pregnant person I think they should introduce vaccines for pregnant women because we are most vulnerable to contract the disease.”

Healthcare workers generally also had high risk perception of COVID susceptibility and severity, particularly in the context of their occupational risk exposure. Male family members did not comment on risk perception related to pregnant and lactating women, and gatekeepers generally had high perception of COVID susceptibility and severity for themselves.

### 3.6 Individual & group influences: Role of the healthcare worker

The role of the healthcare worker emerged as an influence in the decision-making process. While pregnant and lactating women, and male family members and gatekeepers may turn to a healthcare provider for their recommendation about the vaccine, there was no consensus among healthcare workers related to recommending the vaccine.

A major issue was the disconnect between a healthcare worker’s behavior and the recommendation of the vaccine to others, as articulated by this pregnant woman from an urban community in Kakamega: “Even some of the doctors will tell you they have not been vaccinated so you wonder how they will vaccinate me if they have not been vaccinated. You find they are yet to be vaccinated.” Healthcare workers themselves may not recommend the vaccine, including this worker from a rural community in Kakamega: “I have heard that the vaccine is contraindicated in pregnant woman, as the fetus is still young. Maybe it can cause some problems to the fetus, so I will not recommend.” Similarly, this worker from an urban community in Nairobi did not feel comfortable recommending the vaccine: “As for me, I wouldn’t recommend the vaccine, as it is something I am not sure about.” Others were recommending the vaccine to pregnant and lactating women, such as this worker from an urban community in Kakamega: “I do recommend, in fact I normally recommend to at least be vaccinated because at the moment we have this pandemic.” Healthcare workers did see how their recommendation influenced pregnant and lactating women, including this worker from an urban community in Nairobi: “It is only that when they are asking, for one you can read from their eyes, they want you to say that they should be vaccinated, because of information that a pregnant mother should not be vaccinated. As I had told you, when somebody comes to inquire something from a worker, it is like a checklist, she wants you to say that it is ok. If you tell them that it is ok, they will take it, so by telling them that they can get the vaccine, the facial expression that you get from them is that of happiness, it is like they are satisfied and they are comfortable to get it.”

Male family members stressed that they did look to healthcare workers related to recommending the vaccine to pregnant and lactating woman, as seen by this male family member from a rural community in Kakamega: “If the health workers have said that it’s safe then they should be given to pregnant women…if it will be safe for the baby and mother then they should be given and it should be authorized by the Ministry of Health and they know that it’s safe for the child and mother. If it’s not safe then they shouldn’t be given.” This notion was supported by gatekeepers as well, as stated by this gatekeeper from a rural community in Garissa: “We vaccinate our pregnant women as per doctor's directions when we take them to hospitals, if the doctor says that she needs a vaccine…we get vaccines as required.” This gatekeeper from a rural community in Garissa also noted the influence of healthcare workers in the vaccine recommendation: “My opinion is that they should not be given because this is a pregnant mother and as doctors have told us in the past a pregnant mother should not be given strong medication because this child will die. For the health of this child and its mother they should not be given.”

### 3.7 Vaccine & vaccination issues: Availability, accessibility, and eligibility

Three key issues related to the vaccine and vaccination emerged. Accessibility to getting the vaccine, lack of clarity related to eligibility, and vaccine availability were raised as critical to vaccine concerns.

Pregnant and lactating women suggested that the vaccine should be delivered with accessibility in mind. This pregnant woman from an urban community in Nairobi recommended door-to-door campaigns: “The vaccines…I would wish them to be brought door-to-door because people are not volunteering to go where it is.” This lactating woman from an urban community in Nairobi felt similarly: “The vaccine, to get to every-one, it should be like polio. When you are in the house they come and look and if anyone hasn’t been given polio they are given the vaccine.” Eligibility was also a barrier, as this pregnant woman from an urban community in Nairobi elucidated: “I have not been told anything, as a pregnant mother and I do not know if I should get the vaccine or not. I do not know.” This lactating woman from an urban community in Kakamega echoed this sentiment: “But for us lactating mothers, I have wished for myself but I have not yet confirmed if I am eligible for the vaccine.”

Healthcare workers also brought up accessibility, such as this worker from an urban community in Nairobi: “I think that the government is complaining that people are not going for the vaccines, but Kenyans are hustlers, they are people who have to work hard so that they can put food on their table. You want me to come and queue from 8am-4 pm to get a Covid vaccine? They should make sure that they have many vaccination points which are easily accessible, for quick service delivery.” This was also raised by this worker from an urban community in Kakamega: “Regarding maternal immunization, basically immunization is done in the hospital, but most of the pregnant women - they cannot access health care.” Structural barriers, such as cost, were frequently raised related to maternal vaccination, including by this worker from an urban community in Nairobi: “Why are some patients not coming even for the tetanus vaccines, it is because of the distance…they cannot afford transport.”

Cost was also relevant in the eyes of male family members and gatekeepers, including this male family member from a rural community in Garissa: “They need to have money and to someone who is struggling to make ends meet, the husband fends for money to eat that day and nothing is left.” This gatekeeper from a rural community in Kakamega stated: “A person will spend 300 shillings (approximate $3 US dollars) going to get the vaccine. The casual workers in the villages earn 150–200 shillings. If you tell him to go to get the vaccine, he will see that as a luxury.”

## 4 Discussion

Using the SAGE Vaccine Hesitancy model, this study outlines results that provide insight into potential factors affecting COVID-19 vaccine decision-making process among pregnant and lactating women in Kenya, including contextual influences, individual and group influences, and vaccine and vaccination specific issues.

Safety was identified as a top barrier, and in particular, concerns related to fertility. This is in line with other studies conducted in Kenya [28] and the broader region [1], [7]. While risk perception was high, pregnant and lactating women looked to healthcare workers to inform their decision-making process, but our results indicate that healthcare workers themselves have safety concerns, similar to other studies in the region [3], [27]. Addressing healthcare worker hesitancy will be critical to affect vaccine acceptance, given their status in these communities. Identifying specific training and information needs related to vaccine risks and benefits, so that healthcare workers are able to engage in difficult discussions with hesitant clients has shown promise in reducing healthcare worker hesitancy [36], along with strengthening trust between healthcare workers and health authorities [29].

Participants illustrated that many myths abound related to the effects of the vaccine; COVID-19 vaccine misconceptions are not unique to our results [2]. Given the dynamic nature of the pandemic and social media platforms fueling misinformation, to combat and dispel these myths, using culturally and linguistically proficient communication strategies, which involve socio-cultural influencers, engaging community leaders, and targeting vaccine rumors will be necessary [12]. Norms, specifically how male members felt toward the vaccine, affected the decision-making process. Engaging with social networks and family members may be a useful strategy for gaining support for vaccination during pregnancy and increasing awareness, as targeted community education and engagement have been found to be a useful strategy in multiple lower-income countries [4], [30], [34].

Our participants identified vaccine availability, accessibility, and eligibility as barriers. To improve delivery, others have suggested meaningful community involvement, such as including key community stakeholders in the structure and modalities for vaccine delivery [2]. In a study including participants from 24 African countries, the authors suggest that vaccinating through door-to-door approaches or through workplace vaccination programs and thereby addressing these “last-mile issues” could drastically reduce vaccine hesitancy [9].

This study is not without limitations. This qualitative study was not designed to be generalizable, and social desirability bias is likely. Findings were heavily dependent on the cross-sectional nature of the study: data collection occurred during a period when COVID-19 cases and deaths were starting to decline in Kenya. Additionally, during data collection, the Kenya Ministry of Health issued a press statement that stated that pregnant and lactating women could choose to receive a COVID vaccine after counseling, which differed substantially from the previous recommendation (from February 2021), which stated that pregnant and lactating women were not eligible for COVID vaccination.

Despite these limitations, this study has several strengths. This is one of the few studies that have examined the vaccine decision-making process specific to pregnant and lactating women. This study also used a holistic approach to understand the decision-making process among this audience, as interviews with the critical audiences that influence pregnant and lactating women were conducted.

Maternal immunization can substantially reduce the effect of infectious diseases in mothers and infants. But for this approach to be realized and successful, communication to the public is essential, specifically communication that understands and addresses the concerns of pregnant and lactating women. Maternal immunization key stakeholders, including individuals from the government, civil society, faith-based organizations, and development partner agencies need to design interventions that target multiple factors, rather than individual level factors that influence the decision-making process of pregnant and lactating mothers to increase maternal immunization uptake. To inform communication to create demand, contextualized vaccine hesitancy research is critical for ensuring the effectiveness, efficiency, and equity of vaccination services in the region [38]. The research should investigate how people think, feel, and act in relation to a vaccine when developing strategies to generate vaccine acceptance. COVID-19 vaccine communication strategies need to form part of broader trust-building measures that focus on relationships, transparency, and participation.

## Funding

This study was funded by the Bill & Melinda Gates Foundation (grant INV-016977), to R. Limaye/R.Karron at the (Johns Hopkins Bloomberg School of Public Health).

## Declaration of Competing Interest

The authors declare that they have no known competing financial interests or personal relationships that could have appeared to influence the work reported in this paper.
